# Association Between Sleep Duration and Atrial Fibrillation: A Narrative Review

**DOI:** 10.7759/cureus.64147

**Published:** 2024-07-09

**Authors:** Guncha Shaikh, Rutvik Raval, Hiba Shahid, Moitreyo Pandit, Abhinav Kumar, Maira Khalid, Asad Ullah Khalid, Samreen Shaikh, Naima Rahim, Mohamed Mustafa Albshir

**Affiliations:** 1 Medicine, Teaching University Geomedi LLC, Tbilisi, GEO; 2 Internal Medicine, B.J. Medical College, Ahmedabad, IND; 3 Internal Medicine, Liaquat National Hospital and Medical College, Karachi, PAK; 4 Internal Medicine, Nottingham University Hospitals NHS Trust, Nottingham, GBR; 5 Internal Medicine, Patna Medical College & Hospital, Patna, IND; 6 Internal Medicine, Indus Hospital & Health Network, Karachi, PAK; 7 Internal Medicine, National Institute of Cardiovascular Diseases, Karachi, PAK; 8 Research, Faculty of Medicine, Ivane Javakhishvili Tbilisi State University, Tbilisi, GEO; 9 Internal Medicine, Institute of Applied Health Sciences, Chittagong, BGD; 10 Internal Medicine, Khartoum Bahri Teaching Hospital, Khartoum, SDN

**Keywords:** heart arrhythmia, stroke, sleep pattern, depression, atrial fibrillation

## Abstract

Sleep duration is a substantial risk factor for several cardiovascular diseases, including atrial fibrillation (AF). Despite much research, the precise nature of the relationship between the amount of sleep and AF remains unclear. This narrative review explores the relationship between AF and sleep duration, looking at genetic, mechanistic, and epidemiological data to explain this association. A U-shaped association (nonlinear relationship or curvilinear association) between sleep duration and AF has been seen, where longer and shorter sleep duration, more or less than seven to eight hours, have been associated with increased AF risk. Multiple mechanisms such as autonomic dysfunction, inflammation, and structural atrial remodeling have been proposed linking sleep disturbances to AF. Moreover, confounding factors such as individual lifestyle, comorbidities, and sleep quality could affect this association. Additionally, the interpretation of study results is further impacted by methodological limitations, including self-reported sleep duration and observational study designs. It is imperative to comprehend the complex relationship between sleep duration and AF to develop effective preventive and therapeutic methods. The main goals of future research should focus on prospective cohort studies with objective sleep metrics, exploring the mechanistic pathways, and comprehensive confounder adjustments that link sleep disturbances to AF. In summary, addressing sleep disturbances may represent one of the novel approaches to AF prevention and management, with potential implications for improving cardiovascular health and reducing AF-related morbidity and mortality.

## Introduction and background

The European Heart Rhythm Association (EHRA) defines atrial fibrillation (AF) as any arrhythmia lasting at least 30 seconds or displaying ECG characteristics of AF. This definition is widely accepted in research and FDA regulations but has not been directly linked to specific AF outcomes [1].

AF onset depends on a susceptible atrial substrate influenced by comorbidities, genetics, and lifestyle factors. Genetic studies have identified 17 signals associated with AF, while established risk factors such as age and lifestyle contribute to atrial remodeling, increasing the risk of AF and cardiovascular complications. Epidemiological studies have connected various risk factors to AF, leading to questions about the implications of genetic research for risk assessment and treatment strategies. Current research focuses on understanding how modifying these risk factors can affect the burden of AF and improve clinical outcomes [2].

A systematic review found that both short and long sleep durations are linked to a higher risk of AF, suggesting a U-shaped association. However, residual confounders such as depression or anxiety disorders, associated with cardiovascular risk, may influence this relationship. Differences in sleep durations among study populations could also be influenced by predisposing factors such as depression. Earlier reports have highlighted similar U-shaped associations between sleep duration and cardiovascular disease, risk factors, and all-cause mortality [3].

The purpose of this narrative review is to explore the association between sleep duration and AF, examining existing literature to understand the relationship between sleep patterns and AF risk.

## Review

Sleep duration and health

Sleep quality and duration have always been crucial in maintaining overall health and well-being. Multiple studies have suggested that a sleep duration of seven to eight hours per day is most favorably associated with optimal health in adults and older adults [4]. The relationship between sleep and mortality was examined. It was seen that there was a 12% higher mortality risk in people sleeping less than six hours per day [5], while there was a 39% greater risk of mortality in individuals sleeping more than eight to nine hours per day [6].

The incidence of cognitive disorders was observed to be low with seven to eight hours of sleep [7]; meanwhile, a decline in cognitive functions was affected with extreme deviation from it (both increase or decrease) [8]. No significant difference has been noted regarding sleep duration and the incidence of depression [5,6]. In middle-aged and elderly women, a higher risk of osteoporosis was seen with sleep duration longer than eight hours [9]. A study was done across multiple studies, which showed that short and long sleep durations, instead of seven to eight hours, posed a higher risk of falls in adults. Also seen in one study was the stronger association of fall in Caucasians (odds ratio (OR) = 1.69, 95% CI: 1.36-2.08) due to shorter sleep as compared to Asians (OR = 1.23, 95% CI: 1.04-1.45) [10]. Multiple studies also reported that workers with shorter sleep (<6 or 7 hours/day) had an increased risk of work-related injuries [11].

The risk of developing cardiovascular disease increases by 11% for every hour of decrease in sleep duration from seven hours per day. Similarly, each hour increase causes a 7% risk of developing cardiovascular disease [12]. Shorter sleep duration was also associated with a higher incidence of hypertension [13]. Cardiometabolic diseases also increase the risk of heart disease [14,15]. In the case of the incidence of developing type 2 diabetes, there was a 9% higher risk with every hour decrease in sleep from seven hours, as compared to a 14% higher risk with every hour of increase [16]. An absolute increase was seen in cases of obesity, with 38% being associated with less than six hours of sleep per day, and an 8% increase with more than nine hours of sleep [5,6].

AF: Etiology and risk factors

AF is the most prevalent sustained cardiac arrhythmia, affecting 1-2% of developed countries' populations, with a projected 25% incidence among 40-year-olds in the EU [17,18]. AF's incidence rises with age, with advanced age as a strong predictor [17]. It carries double the mortality rate of the normal population and a three- to fivefold increased stroke risk [17,19]. AF may decrease cardiac output by 5-15%, potentially leading to cardiomyopathy and heart failure (HF) [19,18]. Symptoms vary, with some individuals being asymptomatic [17,19]. Management involves rate and rhythm control and anticoagulation therapy [17,18]. Improved treatment strategies emphasize early recognition and intervention [19].

AF is influenced by both established and modifiable risk factors, contributing to its development and recurrence. Established risk factors include aging, hypertension, diabetes mellitus, congestive HF, and valvular heart disease [20,21]. In the Framingham Heart Study, advancing age, diabetes, hypertension, congestive HF, and valve disease were identified as independent predictors of AF [21]. Notably, the risk of AF increases with each decade of age, with men having a 1.5 times greater risk compared to women [21].

Lifestyle factors such as obesity, obstructive sleep apnea (OSA), hypertension, and diabetes mellitus play crucial roles in AF pathogenesis. Obesity is associated with structural and electrophysiological changes in the atria, including left ventricular hypertrophy, atrial dilation, fibrosis, and altered conduction velocities, contributing to AF susceptibility [20]. For instance, individuals with obesity have a 30% increased risk of developing AF compared to non-obese individuals [22]. Similarly, OSA induces acute and chronic atrial remodeling, characterized by atrial dilation, fibrosis, and increased autonomic nerve density, thereby promoting AF initiation and maintenance [20].

Understanding the interplay between established and lifestyle-related risk factors is crucial for developing effective preventive and therapeutic strategies for AF.

Evidence linking sleep duration and AF

The association between sleep duration and incidence of AF has been widely studied. Few studies have reported an increased risk of AF with shorter sleep duration, and a few associated it with longer sleep duration.

One study by Genuardi et al. involving 31,079 patients over 16 years analyzed sleep data recorded by overnight polysomnography, which demonstrated the greater prevalence of AF with shorter sleep duration. It identified a 17% increased risk in AF prevalence and a 9% increased risk in AF incidence with a one-hour reduction in sleep duration [23]. Another longitudinal cohort study found sleep disruption to be an important risk for AF, therefore proposing a link between AF and night awakenings as well, which was also reported by a national cohort study [24]. Furthermore, a meta-analysis also demonstrated a 40% increase in the risk of AF incidence due to frequent nighttime awakening, emphasizing on the importance of overall sleep quality [25].

Some studies also have reported longer sleep duration as a risk factor for AF. A prospective cohort study done on a Chinese population with more than 101,000 participants, spanning a median follow-up period of 7.89 years, identified prolonged sleep as an independent risk factor for AF increasing its risk by 50% [26]. Furthermore, in the Physicians’ Health Study, a prospective cohort study involving more than 18,000 US male physicians, analyzing their self-reported sleep duration, a modestly elevated AF risk was shown with self-reported longer sleep duration [27]. Such an association of higher AF risk with both long and short sleep duration indicates a U-shaped link between AF and sleep duration.

Additionally, another healthcare study compared individuals with varying nightshifts and found that exposure to night shifts, both in the current and over a lifetime, was associated with an elevated risk of AF independent of genetic risk factors. This gives us an idea of how altered sleep duration and circadian rhythm can affect our cardiovascular health [28].

In contrast, the Multi-Ethnic Study of Atherosclerosis (MESA) sleep study done to analyze sleep characteristics found no association between sleep duration and AF. However, in this study, a longer duration of slow-wave sleep was identified to increase the possibility of having AF highlighting the importance of sleep stage distribution in sleep health [29].

Additionally, a UK Biobank study conducted with more than 403,000 participants to investigate associations between a healthy sleep pattern and risk of cardiac arrhythmia concluded that individuals with a healthy sleep score (=5) had a 29% and 35% lower risk of developing AF and bradyarrhythmia, respectively, as compared to ones having sleep score of 0-1. This highlights the importance of improving sleep behaviors in order to prevent cardiovascular diseases, including AF, especially in high-risk groups [30].

There are numerous shared risk factors contributing significantly to the onset of both AF and sleep-disordered breathing. These factors comprise age, gender, hypertension, congestive HF, and coronary artery disease [31]. Distinct pathophysiological pathways also exist whereby disordered sleep patterns can predispose individuals to AF including recurrent hypoxia and increased intra-thoracic pressure, resulting in heightened sympathetic activity, oxidative stress, and structural alterations in the atria [32].

One study by Christensen et al. found that a decrease in rapid eye movement (REM) sleep is independently associated with an increased risk of AF incidence among older adults [24]. During REM sleep, there is a high sympathetic tone that suggests that there is an increase in parasympathetic vagal tone in the case of reduced REM sleep [33]. This enhanced vagal tone can stimulate parasympathetic ganglia present in the left atrium, leading to hyperexcitation of the atrial tissue, which can trigger further episodes of AF [34].

In addition, the dysregulated hypothalamic-pituitary axis (HPA) is said to be a possible cause of this association. In people with insomnia, particularly with objective short sleep duration, there is an increase in sympathetic activity, leading to a rise in hormones such as cortisol suggesting increased HPA axis activity and underlying autonomic dysregulation [35]. This further leads to increased heart rate, altered heart rate variability [36,37], and increased atherogenesis [38], and such alterations can eventually lead to the development of AF [39].

Few studies analyzing the effects of reduced sleep duration and sleep disruption have also demonstrated subsequent increases in inflammatory cytokines such as C-reactive protein (CRP) [37], interleukin-6, and tumor necrosis factor-α [40,41], which results in an accelerated atherogenic state [37]. This rise in inflammatory markers can further contribute to the development of AF [38,40-42].

Moreover, reduced oxygen levels during sleep resulting from associated obstructive sleep disorders can activate sympathetic activity, which in turn heightens myocardial oxygen demand [43,44]. Obstructive sleep patterns can also lead to intrathoracic pressure changes, adding to the myocardial perfusion abnormalities and ultimately leading to tissue stretching and remodeling at the pulmonary vein openings, which are recognized as focal sources of AF [43,45].

Confounding factors and limitations

When exploring the relationship between sleep duration and AF, it is essential to consider various confounding factors that may influence the association. Sleep quality is a significant confounder, with poor sleep quality and sleep disorders such as OSA independently linked to AF risk [46,47]. Lifestyle factors such as alcohol consumption, smoking, and physical activity may also confound the relationship, as they impact both sleep duration and AF risk [48,49]. Additionally, comorbid conditions such as depression, anxiety, and chronic pain, often coexisting with sleep disturbances, could contribute to AF development [50,51]. Socioeconomic status and access to healthcare are additional confounders that may affect both sleep patterns and AF risk [52,53].

Despite the increasing literature on sleep duration and AF, several methodological limitations exist. Many studies rely on self-reported sleep duration, which is susceptible to recall bias and misclassification. Moreover, most studies are observational, limiting causal inference and making them vulnerable to confounding. Heterogeneity in AF ascertainment methods, sleep assessment tools, and adjustment for potential confounders across studies also hamper comparability and generalizability. Furthermore, most studies primarily focus on sleep duration alone, overlooking the interplay between sleep quality and other sleep-related variables. Longitudinal studies with objective sleep duration measures, comprehensive confounder adjustment, and consideration of sleep quality are needed to bolster the evidence.

Future research should address these limitations to better understand the complex relationship between sleep duration and AF. Prospective cohort studies with objective sleep measures and rigorous confounder adjustment are necessary to establish causality. Randomized controlled trials assessing the effects of interventions targeting sleep duration on AF incidence could provide valuable insights. Additionally, research exploring the mechanisms linking sleep duration to AF, such as autonomic dysfunction and inflammation, is warranted. A multidisciplinary approach integrating sleep medicine, cardiology, and epidemiology is essential to advance our understanding and improve AF prevention and management strategies.

Clinical implications and recommendations

Sleep disruption, irrespective of obstructive sleep apnoea, is a significant risk factor for AF, potentially attributed to reduced REM sleep. Research on interventions to improve sleep quality could be pivotal in preventing AF, considering its widespread occurrence and significant negative impacts. Consistent associations across multiple data sources highlight the importance of addressing sleep disturbances in understanding AF pathogenesis [24].

Poor sleep quality was linked to an immediate rise in the likelihood of self-reported episodes of AF, with a dose-response pattern showing that increasingly poorer sleep was associated with longer AF episodes the subsequent day. These findings suggest that the quality of sleep might serve as a changeable trigger affecting the near-term risk of a specific AF episode [54]. Unhealthy sleep patterns, such as sleeping for fewer than six hours or more than eight hours per day, have been linked to an increased likelihood of AF, while studies indicate that short-term enforced sleep deprivation negatively affects various bodily systems [3]. Insomnia is consistently associated with AF, emphasizing the importance of maintaining regular sleep patterns to reduce AF risk. Age, gender, and certain health conditions such as peripheral artery disease can influence this association. Healthcare providers should prioritize addressing sleep issues to potentially lower AF risk and improve cardiovascular health [55].

Adhering to a healthy sleep pattern is linked to a reduced risk of AF and bradyarrhythmia, irrespective of conventional risk factors and genetic predisposition. Considering that cardiac arrhythmias serve as early indicators for cardiovascular diseases and may be reversible, emphasizing the significance of maintaining a healthy sleep pattern remains essential for primary cardiovascular disease prevention [30]. Furthermore, sleep apnoea is commonly overlooked in AF patients due to the limitations of standard detection methods, emphasizing the urgent need for improved diagnostic approaches and treatment strategies to address this issue and optimize patient care in the context of AF management [56].

Continuous positive airway pressure (CPAP) shows a significant relative risk reduction in AF recurrence among OSA patients, regardless of other treatments. This underscores the importance of screening for undiagnosed OSA in AF patients and highlights CPAP as a valuable treatment option. Meta-analyses reveal that CPAP not only reduces sleep-related respiratory events but also improves daytime alertness and overall quality of life [57]. Consistent sleep duration determined by genetics protects against HF and may inversely relate to AF. Short sleep duration raises the risk of both AF and HF, but evidence for long sleep duration's protective effect is inconclusive. Larger intervention studies are necessary to confirm the effectiveness of improving sleep in reducing AF and HF incidence [58].

## Conclusions

This comprehensive review delves deep into the intricate relationship between sleep duration and AF, highlighting the multifaceted nature of this association. It underscores the importance of considering various factors, including sleep quality, lifestyle, comorbidities, and socioeconomic status, in understanding the complex interplay between sleep and AF risk. The evidence presented suggests a U-shaped relationship between sleep duration and AF, with both short and long sleep durations associated with an increased risk of AF. Additionally, the review discusses potential mechanisms underlying this association, such as autonomic dysfunction, inflammation, and structural changes in the atria. Despite the wealth of evidence, methodological limitations and confounding factors necessitate further research to elucidate causal relationships and develop targeted interventions for AF prevention and management.
